# Consciousness, brain, neuroplasticity

**DOI:** 10.3389/fpsyg.2013.00412

**Published:** 2013-07-10

**Authors:** Jean Askenasy, Joseph Lehmann

**Affiliations:** ^1^Sackler Faculty of Medicine, School of Medicine, Tel Aviv UniversityTel-Aviv, Israel; ^2^Faculty of Humanities, School of Philosophy, Tel Aviv UniversityTel-Aviv, Israel

**Keywords:** consciousness, neuroplasticity, memory

## Abstract

Subjectivity, intentionality, self-awareness and will are major components of consciousness in human beings. Changes in consciousness and its content following different brain processes and malfunction have long been studied. Cognitive sciences assume that brain activities have an infrastructure, but there is also evidence that consciousness itself may change this infrastructure. The two-way influence between brain and consciousness has been at the center of philosophy and less so, of science. This so-called bottom-up and top-down interrelationship is controversial and is the subject of our article. We would like to ask: how does it happen that consciousness may provoke structural changes in the brain? The living brain means continuous changes at the synaptic level with every new experience, with every new process of learning, memorizing or mastering new and existing skills. Synapses are generated and dissolved, while others are preserved, in an ever-changing process of so-called neuroplasticity. Ongoing processes of synaptic reinforcements and decay occur during wakefulness when consciousness is present, but also during sleep when it is mostly absent. We suggest that consciousness influences brain neuroplasticity both during wakefulness as well as sleep in a top-down way. This means that consciousness really activates synaptic flow and changes brain structures and functional organization. The dynamic impact of consciousness on brain never stops despite the relative stationary structure of the brain. Such a process can be a target for medical intervention, e.g., by cognitive training.

## Introduction

I am the master of my fate:

I am the captain of my soul

(Henley, 1904/1875).

On Friday evening, at 6:30 pm, on the 4th of August 2006, at the age of 66, while sitting at his table, holding his head with his right hand, I.K. suddenly fell down on his face. He could not move his right leg and arm, neither could he call for help as his ability to speak has been lost. It was later found that his left common carotid artery was obliterated, and stopped supplying blood and oxygen to the left half of his brain. I.K. experienced a disastrous ischemic stroke: within a few minutes almost a quarter of his brain was destroyed, with the dramatic consequences of a paralysis of the right side of his body (hemiplegia), a loss of speaking and comprehension capabilities (aphasia) and inability to write (agraphia).

In this difficult situation, I.K. could still listen to the doctors around him who discussed his situation. At that time, the neurologists could see that I.K. had suffered severe structural damage and had little hope for his recuperation. But, a year later it became evident that I.K. had experienced an unexpected rehabilitation. Four years after his incident, I.K. could already play the piano with his left hand, write books and poems, paint and exhibit his paintings to the public.

What has happened? One potential explanation had to do with the fact that I.K. had an exceptionally developed right hemisphere (the intact one), which had been enhanced by years of playing piano and painting in his free time. No doubt that these activities have played an important role in his surprising recovery. But there is an additional second explanation. In one of his poems written after his incident, I.K. presented his decision to conduct a new form of life: “I want to talk words of wisdom, but I know that my mouth will betray me when I speak … So what is left for me? I have the will to live, not as *I want*, but as *I can.*” This decision was taken despite his dramatic symptoms that included difficulties in writing (dysgraphia), word repetition (perseveration), new words creation (neologisms) and more. It was a conscious decision that represented an act of will and can be considered the source for his astonishing rehabilitation.

Twenty two days after his stroke he created the following picture which shows the paralyzed thumb of his right arm half covered by heads and half empty. The picture demonstrates his struggle to understand his new state. Two years later he painted a city as a structure of chess maintaining houses, entitled by him “Chess board,” image of his rehabilitation.

It is worthwhile to note that it was I.K.'s linguistic, communication, and expressional capabilities that have recovered but less so his motor skills. The secret of this marvelous transformation might have been rooted in his power of will and firm decision to live in the best possible way he can, even though it is severely limited. An involvement of a top-down effect of consciousness on the neuroplasticity of his brain may explain the rehabilitation of I.K., unlike many others in his state.

**Figure:**
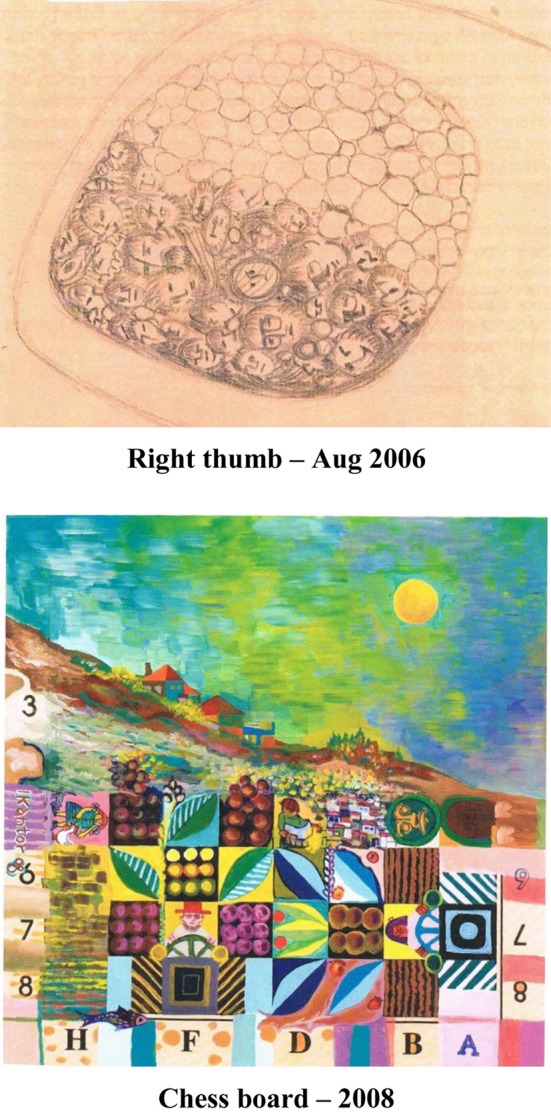


Cases of patient recovery after stroke have been reported by Norman Doidge who wrote in his book:

The human brain can change itself, as told through the stories of the scientists, doctors, and patients who have together brought about these astonishing transformations … Some were patients who had what were thought to be incurable brain problems; others were people without specific problems who simply wanted to improve the functioning of their brains or preserve them as they aged. For 400 years this venture would have been inconceivable because mainstream medicine and science believed that brain anatomy was fixed (Doidge, 2007).

These words imply that consciousness and will can have an important role in brain recovery, but might not be always sufficient for certain kinds of brain damages. Brain recovery is more common in brain which preserve consciousness, memory and cognition. It is less common either in long unconscious states, or in brainstem lesions that are less plastic, have less redundancy, and recover very rarely if at all. When I.K. said: “I want to talk words of wisdom, but I know that my mouth will betray me when I speak” he intuitively recognized this distinction. He felt that his thinking and language capacities might recover, but less so his motor skills that are needed for speaking.

The purpose of this article is to discuss the controversial coexistence of a two-way interrelationship between consciousness and brain biochemistry and neural networks. According to serial and modular bottom-up theories, brain perception extracts features from world reality through the senses. Perception is integrated through higher regions and functions of the brain and may finally involve attention and consciousness.

The philosopher Remo Bodei has suggested that in wakefulness the personality is much more unified and under the control of reason, while in dreaming various personalities tend to exist in parallel in a state of delirium. In other words, a state of consciousness is reached in a converged and ordered mode, achieving gestalt, while a state of dreaming is reached in a diverged and confused mode. Converged mode invites a unified mentalization while diverged mode invites differentiation and plurality. Both of these modes are needed for normal life. In order to translate this concept into a horizontal-vertical axis, the upward movement attempts to ascend to rationality and gestalt, while the downward movement to distributed and fragmented functions. An attempt to draw powers from a unified function to affect distributed functions and structures seems possible if the integrative process can overcome local lesions.

At this point we have to make a distinction between the degrees of gestalt reached by different individuals at a time of injury. According to this view, an interference to bottom-up and top-down processes may vary from one individual to another and influence the elaboration of consciousness. Thought and consciousness are activated by sense-data, but on their turn initiate parallel and interactive processes in the brain where both lower levels (e.g., perception) and higher levels of cognition (e.g., memory, conceptual systems, and world knowledge) occur simultaneously and interactively.

The dynamic nature of the brain is maintained by its neuroplasticity which is closely related to consciousness. In his book, Norman Doidge wrote:

The idea that the brain can change its own structure and function through thought and activity is, I believe, the most important alteration in our view of the brain since we first sketched out its basic anatomy and the workings of its basic component, the neuron (Doidge, 2007).

Vision is a sense that has been researched the most of all senses. During the twentieth century there were major steps that have contributed to our understanding of visual complexity. In 1916, Holmes and Lister discovered the retinotopic distorted mapping in the striate cortex (V1 through V5). Holmes and Lister examined and observed 2000 wounded soldiers, who had devastating brain injuries combined with vision impairments. They documented each of the cases within diagrams of the back of the head, where the position of the wound was represented approximately on a diagram of the back of the head. Horizontal lines represented the distance of the plane above the inion, and vertical lines represented the distance from the middle line of the skull. The location of the wounds were then compared with the character of his blindness for each of the soldiers. A reasonable conclusion was reached, which was that visual information is received in the retina and transferred to the primary visual cortex, where it is kept in the form of an image or representation. Holmes and Lister defined the brain area which is the “cerebral localization of vision, and more particularly … the representation of different regions of the retina in the cortex” (Holmes and Lister, 1916).

**Figure:**
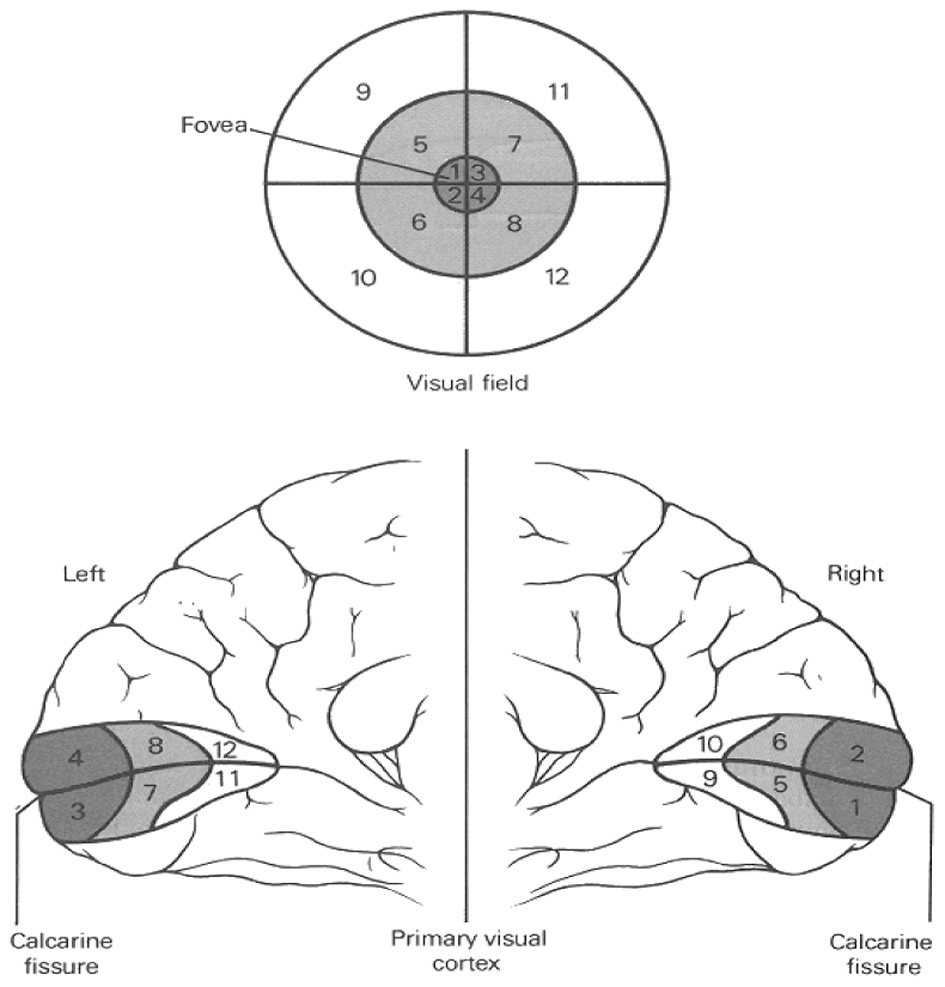


It is a part of visual perception circuits in the brain and support a bottom-up theory where visual information flows through the brain, first perceived in the retina and then integrated and interpreted in the cortex.

In the early years of the 1960th, (Leary and Alpert, 1962) conducted their controversial research on “The psychedelic hallucinogenic effect of drugs” using psychotropic substances (mostly psilocybin mushrooms and LSD). They demonstrated that biochemistry at the synaptic level forms a basis for changes in vision.

In 1965, (Yarbus, 1967) published his book on eye movements and vision which was based on simple methodology, but had followed an extremely perseverant work. Yarbus discovered “the predominant preferences for faces of the brain perception,” which means that visual perception is not a replica of what comes to vision. He also demonstrated that eye movements are task oriented and are different in cases of free observations than in cases of conscious efforts to record specific information from a scene (Yarbus, 1967).

Since Yarbus, task oriented eye movements are commonly referred to as a top-down process where conscious goals can direct low level functions of perception.

**Figure:**
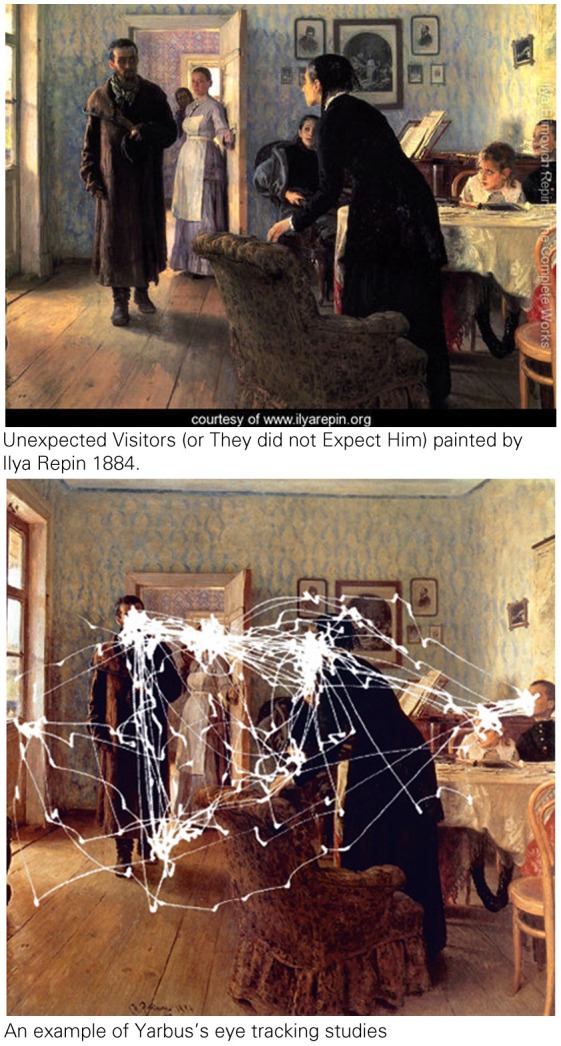


**Figure:**
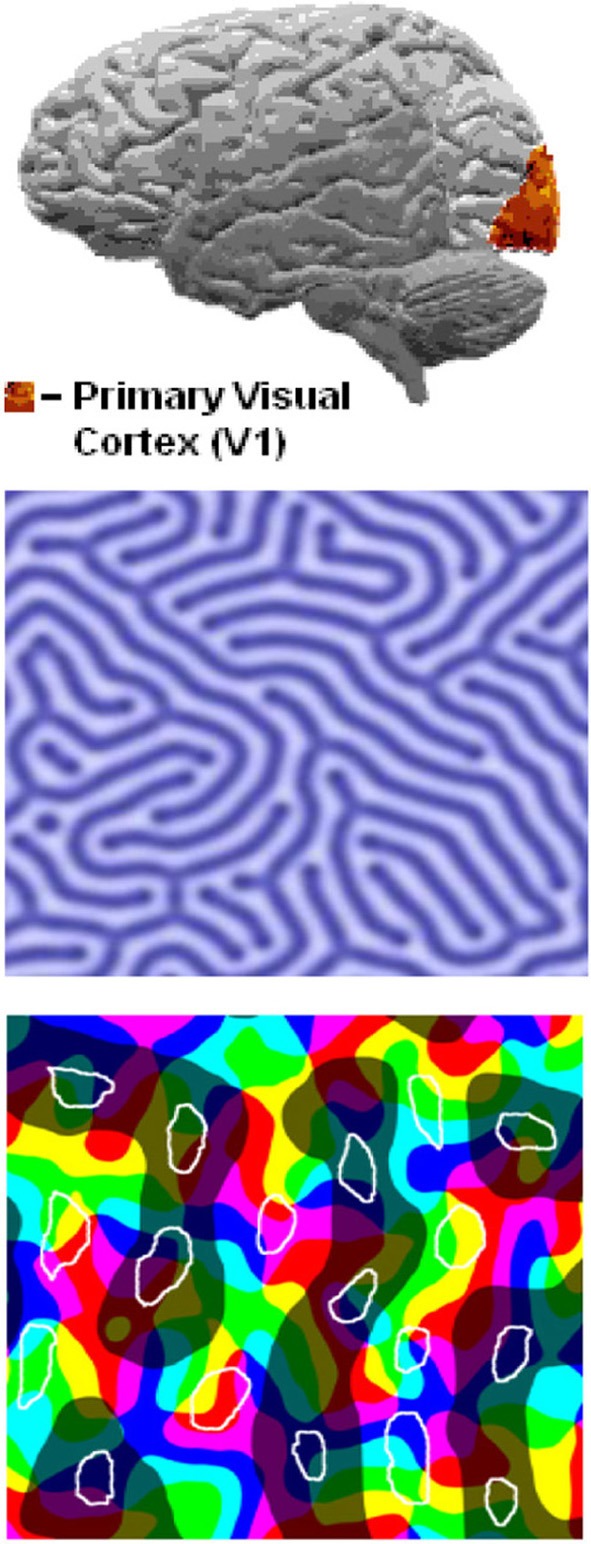


In 1981, Hubel and Wiesel received a Nobel Prize for discovering “The specific orientation of columns and layers perpendicular to the surface of primary visual cortex—V1.”

They could show that the brain of cats can change after they were blinded in one eye. The portion of the cat's brain associated with the blinded eye has changed to process visual information from the open eye. This research contributed to the understanding of neural plasticity of the visual system in the brain that follows experience.

In 1988, Livingston and Hubel described “The visual pathways of form, color, depth, motion and perception.” Their theory showed a bottom-up flow of visual information from the retina, through the optic nerve to the lateral geniculate nucleus, then the primary visual cortex, and finally the extrastriate cortex. Each of these brain areas performs a specific processing of the visual information and provides its output to the next area:

The primate visual system consists of several separate and independent subdivisions that analyze different aspects of the same retinal image … Moreover, perceptual experiments can be designed to ask which subdivisions of the system are responsible for particular visual abilities (Livingstone and Hubel, 1988).

In 1995, Milner and Goodale described “The Visual Brain in Action” with two separate dorsal and ventral streams of visual pathways that have however “multiple connections between them” and “a successful integration of their complementary contributions.” They suggested an unknown interaction among these streams and other parts of the brain that achieves a purpose directed performance of the visual system.

**Figure:**
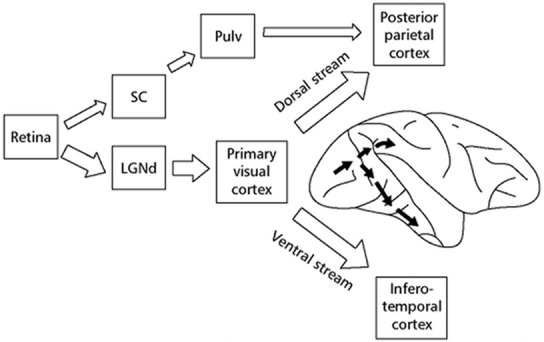
(Source: Milner and Goodale, 1995)

They asked:

How the two streams interact both with each other and with other brain regions in the production of purposive behavior (Milner and Goodale, 1998).

This infers that in order to be able to achieve visual perception of the surrounding world, the whole nervous system has to work within a dynamic structure of connectivity and complexity, which can be termed the “gestalt brain.”

Researchers in Indiana University and Lausanne University Hospital have suggested the term connectome, for a brain network of neurons and their interactions (Sporns et al., 2005). Their suggestion was followed in 2009 by the creation of the Human Connectome Project, whose focus is to build an anatomical and functional network map of the human brain. The Human Brain Project of Henry Markram consists of building a computerized human brain able to simulate a bottom up fashion of artificial brain, in which the top-down effects are not expected to be reached.

Tononi, Edelman and Sporns have responded with a concept of “two fundamental aspects of brain organization” that seem to be in conflict: “the functional segregation of local areas that differ in their anatomy and physiology contrasts sharply with their global integration.” They consider complexity as the major element of the brain vision system and have tried to elaborate methods of measuring it (Tononi et al., 1994).

Dynamic neuroplasticity belong also to the high levels of complexity of the brain circuits. In the visual cortex, new connections are developed after birth and attain their specificity by pruning. Circuit selection depends on visual experience, and the selection criterion is the correlation of activity (Lowel and Singer, 1992). At the highest level of complexity the brain gestalt creates consciousness and the first stage of a top-down pathway is realized. A number of computational models were developed mimicking the gestalt phenomena exemplified by the perception of a visual scene and its segmentation into objects (Wang and Terman, 1997).

The realization of vision at its highest level of complexity is coincident with the achievement of the same level of complexity of other senses, and at this point a certain biochemical constellation characterizes the whole brain.

The disastrous ischemic stroke of I.K., blocked within minutes his brain gestalt. The recuperation process, in the following weeks, made possible the restoration of a certain level of gestalt, which made possible the conscious decision “to conduct a new form of life.” The surprising atypical rehabilitation occurred as a result of the influence of the top-down consciousness effect on brain biochemical constellation, neuronal organization and function.

The initiation of brain processes in the higher brain regions results in an activation of lower brain regions, or the influence of the state of consciousness, on structures of the brain, at bio-chemical and neuroplasticity levels. Neuroplasticity occurs through cellular changes due to learning and memorizing, but also within large-scale changes of cortical remapping in response to injury. Neurogenesis of brain cells can take place in certain locations of the brain, such as the hippocampus, the olfactory bulb, and the cerebellum.

We would like to suggest that the content of consciousness can change without sensorial inputs, as is the case in dreaming. Changes in memory involve continuous structural changes in the brain, both during wakefulness and during sleep. This does not entail a dualistic approach to consciousness and brain but rather another aspect of the living brain as also suggested by Tononi, Sporns, and Edelman. We would like to suggest that top-down and bottom-up processes of consciousness and brain are two aspects of the same complex dynamic and plastic nervous system.

## Consciousness, brain, interaction

But the will is by its nature so free that it can't ever be constrained. … And the activity of the soul consists entirely in the fact that simply by willing something it brings it about that the little gland to which it is closely joined moves in the manner required to produce the effect corresponding to this volition (Descartes, 1649/1985, Vol. I: 343).

But when the soul uses its will to determine itself to some which is not just intelligible but also imaginable, this thought makes a new impression in the brain, and this thought is not a passion in it, but an action which is properly called imagination (Descartes, 1645/2007: 119).

The famous philosopher is most known for his definition of a human being as an entity composed of a thinking mind and a physical body. Descartes was puzzled by the impact of the will and thought on the brain, which seems to contradict the laws of physics. He was of the opinion that the will can result in thoughts that are based both on sensorial experiences and self creative imagination, and that thoughts can actually move the brain. Since his time, the Cartesian mind have been replaced by a more modern concept of consciousness and the brain has took the place of the body to form the “hard problem of consciousness” as defined by the philosopher David Chalmers (Chalmers, 1995). It has been often said that consciousness is an illusion (e.g., Dennett, 1992) and so is free will (e.g., Wegner, 2002). Every event in the world is caused by other prior events in compliance with the laws of nature. And according to the laws of nature, states of the brain cause human thoughts and feelings. This approach is consistent with the natural sciences such as physics and chemistry and denies that an immaterial substance without a mass can cause a material substance to move directly or indirectly. By the same token, traditional neuroscience has defined that a destruction of a major portion of the left hemisphere of the brain in a right dominant person should result in the elimination of all language capabilities independent of any conscious act of will (Popper and Eccles, 1977).

But, writes philosopher John Searle, to accept a scientific view of consciousness does not entitle that consciousness does not exist. In fact, he thinks that both the brain and consciousness do exist, while consciousness is a higher level view of the brain:

- Conscious states, with their subjective, first-person ontology, are real phenomena in the real world.
- All forms of consciousness are caused by the behavior of neurons and are realized in the brain system, which is itself composed of neurons.
- Conscious states … exist at a level higher than that of neurons and synapses. Individual neurons are not conscious, but portions of the brain system composed of neurons are conscious.
- Because conscious states are real features of the real world, they function causally.
- Consciousness is a system-level, biological feature (Searle, 2004: 112–115).

According to Searle, there is no problem in changing consciousness and by that causing the brain to change. A change can be made in the highest level of the brain system or in its lowest level, and the results will include changes in both neurons and consciousness.

We consider consciousness as the result of the growing complexity of connectome activities. Loss of consciousness due to a damage to the cerebrum is often recoverable, while loss due to damage to the brainstem is not. Major damages to the brain may be recovered and consciousness regained through the brain capacity for physical and functional change. However, when there is a vast damage to the brain and the brainstem (the major pathway from the external world to the internal world), consciousness is completely and permanently lost. Brain death and unrecoverable coma with unconsciousness are among these terminal cases, in which neuroplasticity is mostly absent. We can conclude that consciousness favors brain plasticity.

Block and MacDonald (2008) consider two types of consciousness. A phenomenal consciousness that goes beyond cognitive accessibility and a narrower access consciousness “a subject can have an experience that he does not and cannot think about.” Phenomenal consciousness consists of rich experience and feelings, and only part of it reaches thoughts. “although much of the detail in each picture is phenomenally registered, it is not conceptualized at a level that allows cognitive access.” Access consciousness consists of information held in the cognitive system for the purposes of reasoning. Accordingly, there is a more localized core neurological basis for phenomenological consciousness, and there is a broader total neural basis which initiates a level of access consciousness, e.g., a level that involves abstract concepts and language. The core neurological basis can interact with the total neural basis in both a bottom-up and top-down fashions.

Block and MacDonald think that the brain records experiences within a phenomenal consciousness in core neural bases of consciousness. They provide an example of the fusiform face area, at the bottom of the right temporal lobe in the brain, which is activated with a visual experience of faces, and might be regarded as the core neural basis of face perception. Not like phenomenal consciousness, access consciousness involves the whole brain or the gestalt brain, which Block and MacDonald call the total neural basis of consciousness. Gestalt brain includes working memory, attention, high level information processing and integration, rationality, intentionality and introspection, achieving a high state of consciousness which can in turn influence changes in specific parts of the brain structures and functions, language, thoughts and reports (Block, 2005; Block and MacDonald, 2008).

What can be said of access consciousness as a function of the whole brain? It is essential to the sense of the self. It can include imagined objects and events independent of having experience. It is capable of creative and seemingly uncaused function. That is, it can initiate a chain of events without identifiable causes. We have many evidences that consciousness can cause physical changes. It has features such as intentionality and purpose that are hard to explain by past events. It can change itself and it has a self healing capacity which means that in general it can both improve and cure itself—at least to a certain extent. These capabilities of consciousness have been utilized in rehabilitation plans for patients who suffered brain strokes, or for improvement of attention and memory decline in old adults by cognitive training programs (Smith et al., 2009).

Levine (1983) has introduced the term “the explanatory gap” and later Chalmers (1995) has coined “the hard problem of consciousness,” both expressed the opinion that consciousness has a subjective basis that can not be observed and experienced by a third party neither can it be explained by reductive methods—namely by empirical sciences. According to them, it is possible to describe processes in the brain in a scientific objective way, based on observations, but it is not possible to describe personal experience in such a way.

Even without a scientific explanation, the interrelationship between consciousness and brain activity exists in both senses, the observable and the subjective. The paradox of consciousness arises from the fact that even though we do not have a scientific description and explanation of access consciousness and subjectivity based on brain structures and functions, there are still clear evidences of correlation and even causal relations between them. Using Block and MacDonald's concepts of consciousness we can conclude that the content of consciousness can be changed by experience, but also outside of experience.

## Consciousness, brain, and language

At the center of research of both human brain and cognition stays language. Consciousness has an interesting and important relationships with language. Thoughts can be changed by language, sounds, articulation, learning, written text, concepts and associations. Human beings use language all the time for both communication and for thinking. Language provides a frame for beliefs and presumptions and enables deliberation. The interrelationship between thoughts, language and brain structures and functions has been demonstrated by fMRI studies.

fMRI demonstrates that language effects areas in the brain. Different parts of the brain may be activated by different linguistic content. For example, emotional text can cause activity in parts of the brain that have to do with emotions within the limbic system, such as the amygdala, while decision process may activate other areas such as the prefrontal cortex. Language activates Broca's area for articulation and Wernicke's area for comprehension, both reside in the left brain hemisphere. Some of the linguistic activities that take place in wakefulness stay with us and become stable in the form of long term memories.

The interrelationship between the content of consciousness and brain activity as demonstrated by language seems to be concomitant. But how are experiences, thoughts and other mental events recorded in the brain? What happens when we study a new language or develop our knowledge and skills of our first language? Such activities start with consciousness and some brain activity (as can be observed by fMRI and other imaging technologies), and with acquisition of new short term explicit memories, that may later become long term and implicit through farther changes in the brain. On their side, practicing syntactic, semantic and pragmatic aspects of language, and knowledge acquisition might be accompanied by the development of automatic unconscious performance of new skills and knowledge. Still later, changes in the brain can also reverse the process, i.e., erase memories and eliminate past knowledge, including linguistic knowledge of words and their meanings. Language, consciousness and the brain change together dynamically and interactively, in ways that can be regarded as both bottom-up and top-down. Within this interaction, language is used as a cognitive tool, but it also forms a constitutive part of cognition itself (Dascal, 2002).

There is an inherent tension between the bottom-up approach which explores the brain in an analytic and modular way and strives to explain the brain, and from it consciousness, by an ensemble of neuron cells and their associated biochemical processes, and a top-down approach that considers the brain as a complex ever changing living system with integrated and interactive parallel functions that appear in the form of consciousness.

Neuroscientist Eric Kandel has made a distinction between implicit memory that is acquired involuntarily “from the bottom up” and assists automatic forms of response to stimulation, and “spatial memory” which serves consciousness and is the result of willful “top-down” registration of new memories in the brain hippocampus, a process triggered by voluntary attention originated in the cerebral cortex.

Language is at the center of both bottom-up and top-down approaches. A bottom-up approach would require a physiological localization of language faculty that would go from sounds and phonemes, and all the way to meanings of words, sentences and understanding of human discourse. Being connected to both the public world and to the private thought, it is a bridge between the physical and the mental. Localization efforts to find the physical parts that establish mental functions in the brain were initiated by neuroanatomist Franz Gall more than 200 years ago. The search for a speech center in the brain made considerable progress in the 19th century, with the findings of Paul Broca who worked with aphasia patients and defined the so-called Broca's area as responsible for articulated language. A different opinion was presented by his contemporary neurologist John Jackson who thought the Broca's area could block articulation and be essential for it, but is only a link in a chain that involves the whole brain. More recently philosopher Karl Popper and neurophysiologist John Eccles have jointly expressed their opinion that consciousness and brain interact through a speech center within the left cerebral hemisphere of the brain (Popper, 1994; Popper and Eccles, 1977). But the real existence of such a speech center or even the allocation of speech articulation, hearing, and understanding to specific locations in the brain is still controversial.

Schnelle (2010) suggests a three fold research into language in the brain to try and understand the relations between language and brain. He suggests a combined study of linguistics, psychology (which he says is the phenomenological study of the mind), and neurocognitive science (to which he refers as biology). A new interdisciplinary triangle of interdependent perspectives results from the joint consideration and comparison of formal structures, conscious phenomenological images, and brain architecture, and their functions in language. The idea of Schnelle is to look for basic brain networks and structures that represent pieces of knowledge or memories, and try to relate them to language. Such brain functional neural networks are distributed over relatively large areas within the brain and are termed “cognits” by him. He cites Joaquin Fuster who defined a relation between cognition and brain networks as follows:

Cognitive functions, namely perception, attention, memory, language, and intelligence, consist of neural transactions within and between these networks (Fuster, 2006).

Schnelle adds that pieces of knowledge, i.e., cognits, are embedded in neural networks largely through language hearing and speaking and later also reading and writing which guide imagination and abstract structuring. Fine-tuning of brain networks will influence both the automatic non-conscious language production, and the conscious articulation of ideas, plans and knowledge systems of thoughts. Instead of looking for specific static neuron structures in the brain, we should look for dynamic (plastic) distributed networks, which form pieces of linguistic knowledge (cognits), and for their interaction to form a cognit complex. In a way, dynamic conscious ideas cause changes in the brain.

One important cognitive resource to establish both cognition and cognits is memory. It is possible to examine changes in consciousness and the brain through the prism of dynamic memory, recording and forgetting. Short term memory or working memory is so called because of its vulnerability—it does not hold memories for long and presumably has a minor permanent effect on the brain. Long term memory is more durable and involves permanent changes in the brain through creation of proteins, and formation and dissociation of neural networks (Kandel, 2006). We now turn to examine how long term memory develops and changes during sleep.

## Consciousness and brain during sleep

So long as the mind is joined to the body, then in order for it to remember thoughts which it had in the past, it is necessary for some traces of them to be imprinted on the brain; it is by turning to these […] that the mind remembers. So is it really surprising if the brain of […] a man in a deep sleep, is unsuited to receive these traces? (Descartes, 1649/1985, Vol. II: 247).

Descartes believed that memories are formed from thoughts during conscious wakefulness only. Memory encoding and retrieval take place most effectively during wakefulness (Diekelmann and Born, 2010), but sleep also promotes the consolidation of newly acquired information in memory and its integration within pre-existing knowledge networks (Karni et al., 1994; Askenasy et al., 1997). Memory consolidation during sleep is often considered as an off-line brain process of stabilization of such newly acquired information, but there is also consolidation of false memories of events that never happened. Acquired information is transformed, restructured, abstracted, integrated with previously acquired memories, prioritized according to its emotional significance, distorted, inferred and combined with false memories within the process of memory consolidation during sleep (Payne et al., 2009).

Memory consolidation involves structural changes in the brain. The creation of proteins, changes in neural pathways and the creation, destruction, enhancement, and regress of neuronal synapses which are parts of the dynamic neuroplasticity of the nervous system. It is an important phase in learning of both procedural (how to do) and declarative (about) knowledge. Long term memories can become implicit, automatic and uncontrolled such as in riding bicycles, and explicit such as records of events and facts. Consciousness is essential for an episodic and explicit memory acquisition. When consciousness is abolished as happens during coma or epileptic seizures memory acquisition and its associated brain processes stop. It can be inferred that neuroplasticity is a physical quality of the nervous system that can be caused by changes in consciousness, which by themselves can be caused either by explicit sensorial inputs or implicit internal states of mind.

Sleep has been considered by Diekelmann and Born as a state where behavioral control and consciousness are both lost. However, dreams can be recalled upon waking-up. World events such as loud noises can be monitored while sleeping, they can participate in dream developments, and can interrupt sleep. Some forms of learning and post-learning as well as problem solving continue during sleep. Post-learning sleep not only strengthens memories but also induces qualitative changes in their representations and so enable the extraction of invariant features from complex stimulus materials, the forming of new associations and, eventually, insights into hidden rules (Diekelmann and Born, 2010). It is evident that some form of consciousness exists in this state.

Dreams are considered as a mixture of false and true events, at least partially caused by consciousness and result in real changes in the brain such as the formation of new neuron networks. Diekelmann and Born (2010) present a view that memory systems compete and reciprocally interfere during wakefulness, but disengage during sleep, allowing for the independent consolidation of memories in different systems. Nir and Tononi consider dreaming to be a state where:

Human brain, disconnected from environment, can generate an entire world of conscious experiences by itself, The dreamer is highly conscious (has vivid experiences), is disconnected from the environment (is asleep), but somehow the brain is creating a story, filling it with actors and scenarios, and generating hallucinatory images (Nir and Tononi, 2010).

The common denominator of many theories trying to explain dreams is their implicit perception. Such theories include the cognitive theory of dreaming of Hall, who stated:

The images of a dream are the concrete embodiments of the dreamer's thoughts; these images give visual expression to that which is invisible, namely, conceptions (Hall, 1966).

And the activation-synthesis hypothesis of Hobson and McCarley which suggested an automatic and periodic brain stem neuronal mechanism, which generates and determine the spatiotemporal aspects of dream imagery, which are then compared and synthesized with stored memories (Hobson and McCarley, 1977).

In order to analyze the brain mechanism of dreams and hallucinations, we have to switch from sensorial perception to extrasensory perception or from explicit perception to implicit perception. The field where sensorial perception interfere with extrasensory perception is the field of dreams and is different from hallucinations.

The major difference between the two states of sleep and wakefulness is the switch of perception from implicit to explicit. The blocking of implicit perception is reached by the Gestalt brain during wakefulness. During sleep, many parts of the brain show much reduced activity while others continue to be active. Certain sensorial inputs to the brain are disconnected, as well as certain outputs, as muscles and movement control. Consciousness changes and decreases in functions including voluntary control, self awareness and reflective thought. But consciousness also increases in its emotional involvement and impaired memory (dream amnesia) (Nir and Tononi, 2010). Nir and Tononi conclude that “dream consciousness can not be reduced to brain activity in REM sleep,” and that dream is a powerful form of imagination where presumably brain activity flows in a “top down” manner.

In a state of dreaming the external world is almost absent and an internal world exists in the brain and takes over consciousness. The content of dreams is the segregated and integrated external reality deposited in the hippocampus, posterior temporal fusiform gyrus, orbito-frontal area, limbic area, and all over the brain which takes the place of reality. The moment we switch to the wake state the implicit perception is differentiated from the explicit perception and is recognized within consciousness to be a false perception.

The unreal aspect of dreaming is later recognized by the dreamer, and dreams are mostly separated from real experiences. Dreamers are conscious when awakened that the visual imagery that they have experienced was false due to the instant instauration of the Gestalt brain. In the wakefulness state there are normally no self-generated implicit perceptions that are not caused by real experiences. Upon arousal from a dream, consciousness allows the interpretation of the imagery as false. It is a chaotic combination of recollected experiences presented in the mind as occurring now. The ability to experience past implicit visual perceptions characterizes dreams, as the ability to experience explicit perception characterizes visual consciousness during wakefulness.

What differentiate phenomenological explicit from implicit perception are (a) the bizzarness, (b) threatening, (c) vivid colored faces, (d) absence of bodies and limbs, (e) lack of identification and recognition. The switch to wakefulness may be not concomitant with conscious recognition of false imagery, sometimes a longer duration of wakefulness is requiered. Interpretation in wakeness state of the visual imagery as being real means pathology.

Hallucinations are implicit perception, internally generated by the brain, which instead of appearing in the dream state appear in wakefulness, e.g., due to structural lesions of the brain.

During the sleep state a concomitant implicit and explicit perception may occur in lucid dreaming. In the wake state a concomitent implicit and explicit perception may occur in a hallucinatoric brain. In a normal physiologic brain the implicit perception appears as a past experience and is called reminding or remebering. The consciousness of distinguishing the present from the past allows the implicit perception to appear as memory. The fourth element of existence “the time” differentiates memory from implicit perception.

The gestalt waking brain is obliged to separate the implicit from the explicit perception, in order to allow consciousness. If not, the implicit is integrated in the explicit and the extrasensory perception becomes hallucinatory mixture of real and unreal.

The activity of the brainstem and cortical areas during sleep allow an implicit perception to become explicit. The switch to wakefulness may not be concomitant with consciousness of false experiences.

Domhoff has developed a “cognitive theory of dream” that suggests a conceptual system which forms the basis for knowledge and beliefs. During wakefulness it serves for thought and imagination. In sleep it is active within the cortical mature neural network, when external stimuli are blocked and conscious self-control is lost:

A “dream” is a form of thinking during sleep that, as already briefly stated, occurs when there is (1) an intact and fully mature neural network for dreaming; (2) an adequate level of cortical activation; (3) an occlusion of external stimuli by the sensory gates located in the thalamus; and (4) the loss of conscious self-control, i.e., a shutting down to the cognitive system of “self.” … a “dream” is also what people remember in the morning, so it is in this sense a “memory” of the dreaming experience (Domhoff, 2010).

Creativity can be based on autonomic brain events that are initiated in higher brain parts which do not result directly from experience and information that might otherwise be received from lower brain parts. A discontinuity of brain structures which can be accompanied by separate processes and on-going brain changes, together with the capacity of imagination and dream recollection as initiated within the brain during different states of consciousness, may be the basis for an “explanatory gap” and a “hard problem of consciousness.” Top-down and bottom-up processes may be occasionally disconnected leading to a possibility of independent psychological reality, that is not directly caused by sensorial inputs, or otherwise emerge from such inputs in a complex delayed and dynamic ways that can not be inferred from direct observations of lower brain processes.

John Searle makes the same point. He writes that a dream of something red may involve a change in the content of consciousness that is not based on experience but is created in the brain:

Like many people, I dream in color. When I see the color red in a dream, I do not have a perceptual input that creates a building block of red. Rather the mechanisms in the brain that create the whole unified field of dream consciousness create my experience of red as part of the field (Searle, 2004: 155).

## Summary and conclusions

A high-level phenomenon is weakly emergent with respect to a low-level domain when the high-level phenomenon arises from the low-level domain, but truths concerning that phenomenon are unexpected given the principles governing the low-level domain. Weak emergence is the notion of emergence that is most common in recent scientific discussions. A high-level phenomenon is strongly emergent with respect to a low-level domain when the high-level phenomenon arises from the low-level domain, but truths concerning that phenomenon are not deducible even in principle from truths in the low-level domain … I think there is exactly one clear case of a strongly emergent phenomenon, and that is the phenomenon of consciousness (Chalmers, 2006).

Chalmers defines a system property of weak emergence which corresponds to an analytic, bottom-up construction of knowledge. A bottom-up process may start with some sensory inputs which affect some neurons, that change and form new networks with different strengths of connections. According to such a scientific view, consciousness, memories and thoughts are the result of a neuronal integrated activity. Cases that might be described with the concept of weak emergence of system properties and simple learning have been studied in simple organisms such as the Aplysia californica (Kandel, 2006). One day, it is said, it will be possible to form a dynamic model of neurons, their interconnections and their relative strengths. If achieved, this might result in a conscious behavior or at least as a tool to change and enhance cognition.

Chalmers defines a second concept of strong emergence which requires a high level of system knowledge through a top-down approach to exploration. Chalmers claims that knowledge about consciousness is a case in point, where a bottom-up methods will not do for a complete and detailed explanation. His suggestion follows the ideas of the 17th century philosopher Gottfried Leibniz and those of the 20th century founder of general system theory, the biologist von Bertalanffy (1968). Both promoted the application of a whole system top-down approach to living systems (i.e., animals).

We have suggested in this article that dreams include self generated brain images and are results of implicit perception. They are formed by brain networks in the gestalt brain through the activity of neurons and synapses already consolidated and generating false memories integrated with true memories. In this way dream can change the brain by the activation of relatively large neuron ensembles, eliberated from the consciousness control. We can farther speculate that thinking, moral judgments, complex learning (i.e., language acquisition), and many other mental activities, require a concept of strong emergence of consciousness and top-down brain processes. There are other similar states of affairs that are quite common in biology, e.g., swarm behavior.

The neurophysiologist Sir John Eccles and the philosopher Sir Karl Popper had cooperated to find explanations for consciousness, brain and the self. Popper identified the self with full consciousness which, according to him, controls human action.

Full consciousness … consists mainly of thought processes … The self, or full consciousness is exercising a plastic control over some of our movements which, if so controlled, are human action … The novel structures that emerge always interact with the basic structure of physical states from which they emerged … Mental states interact with physiological states (Popper, 1994: 115, 132).

Eccles introduced his hypothesis of interaction between self-conscious mind and the brain. He presented a hypothesis that the unity of consciousness is provided by itself rather than by neural cells in the brain. He went as far as making the following statements:

The self-conscious mind exercises a superior interpretative and controlling role upon the neural events. The unity of conscious experience is provided by the self-conscious mind and not by the neural machinery (Popper and Eccles, 1977: 355).

The traditional scientific bottom-up methodology concentrates on the analysis of modular substructures and biochemical processes within the brain. A top-down concept elaborates a diverged activity from consciousness and cognition to various structures and functions of the brain. Top-down research has concentrated more on high level study of consciousness and cognition, and has gained its own place by taking a system approach to questions on brain and consciousness. John Searle has a similar observation:

Most researchers adopt the building block approach … It seems very difficult to try to study massive amounts of synchronized neuron firings that might produce consciousness in large portions of the brain such as the thalamocortical system … I am betting on the unified-field approach (Searle, 2004: 155–156).

We have followed the above thinkers by highlighting some facts and evidences that can shed light on these two antithetic approaches. We have tried to view consciousness and brain interaction through an instance of exceptional recovery from a broad and dramatic damage to the brain, and by a demonstration of acquired evidences of neuroplasticity in the nervous system through life cycles of wakefulness and sleep. It is evident that consciousness changes the brain while it is also being changed by the brain. Procedural and declarative memory, their acquisition and consolidation are all changes in the nervous system that involve both consciousness and the brain bi-directionally and interactively.

Richard Davidson has been using concepts and phrases such as contemplative neuroscience, neural-inspired behavioral or mental interventions, putting the mind back in medicine, and mental exercise. He and his team have conducted experiments where meditation was used as a conscious mental practice, while consequential changes in the brain were tested with fMRI. Based on his experiments Davidson has suggested that mental training changes the structure and function of the brain:

Mental training of meditation is fundamentally no different than other forms of skill acquisition that can induce plastic changes in the brain (Davidson and Lutz, 2008).

In a later paper he wrote that

Moderate to severe stress appears to increase the growth of several sectors of the amygdala, whereas the effects in the hippocampus and prefrontal cortex tend to be opposite. Structural and functional changes in the brain have been observed with cognitive therapy and certain forms of meditation, we can also take more responsibility for our minds and brains by engaging in certain mental exercises that can induce plastic changes in the brain, it is apparent that both structural and functional connectivity between prefrontal regions and sub cortical structures is extremely important for emotion regulation and that these connections represent important targets for plasticity-induced changes (Davidson and McEwen, 2012).

It is a common experience and has been observed scientifically that the nervous system changes continuously following internal and external events as well as thoughts, imaginations and dreams. Such changes are followed by changes in memory and content of consciousness. It is clear that the damage caused to the brain of I.K. by his stroke changed his consciousness rather dramatically. There is no doubt in our minds that his outstanding brain functions recovery was also the result of his strong will and determination. It was a whole system top-down effect, rather than a property of separate groups of neurons in his brain. This case and other similar cases could lead to a conclusion that medical treatment should target the bidirectional activity of the nervous system, and could better perform if both bottom-up and top-down considerations would be applied in medical interventions, i.e., to add methods that are intended to activate changes in the content of consciousness to more traditional treatments of structural lesions. A combined approach could promote physical and mental health.

### Conflict of interest statement

The authors declare that the research was conducted in the absence of any commercial or financial relationships that could be construed as a potential conflict of interest.

## References

[B1] Askenasy J. J. M. Karni A. Sagi D. (1997). Visual skill consolidation in the dreaming brain, in Sleep and Sleep Disorders: from Molecule to Behavior, eds Hayaishi O. Inoué S. (Tokyo: Academic Press Harcourt Race), 361–376

[B2] Block N. (2005). Two neural correlates of consciousness. Trends Cogn. Sci. 9, 46–52 10.1016/j.tics.2004.12.006 15668096

[B3] Block N. MacDonald C. (2008). Phenomenal and access consciousness. Proc. Aristotelian Soc. CVIII, 289–317 10.1111/j.1467-9264.2008.00247.x

[B4] Chalmers D. J. (1995). Facing up to the problem of consciousness. J. Conscious. Stud. 2, 200–219

[B6] Chalmers D. J. (2006). Strong and weak emergence, in The Re-emergence of Emergence, ed Clayton P. Davies P. (Oxford, UK: Oxford University Press), 244–255

[B7] Dascal M. (2002). Language as a cognitive technology. Int. J. Cogn. Technol. 1, 35–61 10.1075/ijct.1.1.04das

[B8] Davidson R. J. Lutz A. (2008). Buddha's brain: neuroplasticity and meditation. IEEE Signal Process. 25, 171–174 10.1109/MSP.2008.4431873 20871742 PMC2944261

[B9] Davidson R. J. McEwen B. S. (2012). Social influences on neuroplasticity: stress and interventions to promote well-being. Nat. Neurosci. 15, 689–695 10.1038/nn.3093 22534579 PMC3491815

[B10] Dennett D. C. (1992). Consciousness Explained. Boston, MA: Back Bay Books

[B11] Descartes R. (1649/1985). The passions of the soul, in The Philosophical Writings of Descartes. Vol. I, II trans J. Cottingham, R. Stoothoff, and D. Murdoch (Cambridge: Cambridge University Press).

[B12] Descartes R. (1645/2007). The Correspondence between Princess Elisabeth of Bohemia and Rene Descartes. Chicago, USA: University of Chicago Press

[B13] Diekelmann S. Born J. (2010). The memory function of sleep. Nat. Rev. Neurosci. 11, 114–126 10.1038/nrn2762 20046194

[B14] Doidge N. (2007). The Brain that Changes Itself. New York, NY: Viking Penguin

[B15] Domhoff G. W. (2010). The Case for a Cognitive Theory of Dreams. Retrieved December 13, 2012 from http://www2.ucsc.edu/dreams/Library/domhoff_2010a.html

[B16] Fuster J. M. (2006). The cognit: a network model of cortical representation. Int. J. Psychophysiol. 125–132 10.1016/j.ijpsycho.2005.12.015 16626831

[B17] Hall C. S. (1966). The Meaning of Dreams. New York, NY: McGraw-Hill

[B18a] Henley W. E. (1904/1875). Invictus, in The Oxford Book of English Verse 1250–1900, ed Quiller-Couch A. T. (Oxford, UK: Clarendon Press), 1019.

[B18] Hobson J. A. McCarley R. (1977). The brain as a dream state generator: an activation-synthesis hypothesis of the dream process. Am. J. Psychiatry. 134, 1335–1348 21570 10.1176/ajp.134.12.1335

[B19] Holmes G. Lister W. T. (1916). Disturbances of vision from cerebral lesions with special reference to the cortical representation of the macula. Brain 39, 34–37 10.1093/brain/39.1-2.34 PMC2017232 19979442

[B20] Kandel E. R. (2006). In Search of Memory: The Emergence of a New Science of Mind (New York, NY: W. W. Norton and Company).

[B21] Karni A. Tanne D. Rubenstein B. S. Askenasy J. J. M. Sagi D. (1994). Dependence on REM sleep of overnight improvement of a perceptual skill. Science 265, 679–682 10.1126/science.8036518 8036518

[B22a] Leary T. F. Alpert R. (1962). Letter to the Editor. The Harvard Crimson.

[B22] Levine J. (1983). Materialism and qualia: the explanatory gap. Pac. Philos. Q. 64, 354–361 23073546

[B23] Livingstone M. Hubel D. (1988). Segregation of form, color, movement, and depth: anatomy, physiology, and perception. Science 240, 740–749 3283936 10.1126/science.3283936

[B24] Lowel S. Singer W. (1992). Selection of intrinsic horizontal connections in the visual cortex by correlated neuronal activity. Science 255, 209–212 10.1126/science.1372754 1372754

[B25a] Milner D. Goodale M. (1995). The Visual Brain in Action. Oxford: Oxford University Press

[B25] Milner D. Goodale M. (1998). The Visual Brain in Action. Psyche.

[B26] Nir Y. Tononi G. (2010). Dreaming and the brain: from phenomenology to neurophysiology. Trends Cogn. Sci. 14, 88 10.1016/j.tics.2009.12.001 20079677 PMC2814941

[B28] Payne J. D. Schacter D. L. Propper R. E. Huang L. Wamsley E. J. Tucker M. A. (2009). The role of sleep in false memory formation. Neurobiol. Learn. Mem. 92, 327–333 10.1016/j.nlm.2009.03.007 19348959 PMC2789473

[B29] Popper K. R. (1994). Knowledge and the Body-mind Problem. Oxford: Routledge

[B30] Popper K. R. Eccles J. C. (1977). The Self and Its Brain. Berlin: Springer-Verlag

[B31] Schnelle H. (2010). Language in the Brain. Cambridge: Cambridge University Press

[B32a] Searle J. R. (2004). Mind: A Brief Introduction. New York, NY: Oxford University Press

[B32] Smith G. E. Housen P. Yaffe K. Ruff R. Kennison R. F. Machncke H. W. (2009). A cognitive training program based on principles of brain plasticity: results from the improvement in memory with plasticity-based adaptive cognitive training (IMPACT) study. J. Am. Geriatr. Soc. 57, 594–603 10.1111/j.1532-5415.2008.02167.x 19220558 PMC4169294

[B33] Sporns O. Tononi G. Kötter R. (2005). The human connectome: a structural description of the human brain. PLoS Comput. Biol. 1:e42 10.1371/journal.pcbi.0010042 16201007 PMC1239902

[B34] Tononi G. Sporns O. Edelman G. M. (1994). A measure for brain complexity: relating functional segregation and integration in the nervous system. Proc. Natl. Acad. Sci. U.S.A. 91, 5033–5037 8197179 10.1073/pnas.91.11.5033 PMC43925

[B35] von Bertalanffy L. (1968). General System Theory: Foundations, Development, Applications. New York, NY: G. Braziller

[B36] Wang D. Terman D. (1997). Image segmentation based on oscillatory correlation. Neural Comput. 9, 805–836 10.1162/neco.1997.9.4.805 9161023

[B37] Wegner D. M. (2002). The Illusion of Conscious Will. Cambridge, MA: MIT Press

[B38] Yarbus A. L. (1967). Eye Movements and Vision. New York, NY: Plenum Press

